# How can and should we optimize extracorporeal shockwave lithotripsy?

**DOI:** 10.1007/s00240-017-1020-z

**Published:** 2017-11-25

**Authors:** Christian G. Chaussy, Hans-Göran Tiselius

**Affiliations:** 1 University of Munich, Munich, Germany; 2 University of Regensburg, Regensburg, Germany; 3 Keck School of Medicine, USC, Los Angeles, USA; 4 Division of Urology, Department of Clinical Science, Intervention and Technology (CLINTEC), Karolinska Institutet, Stockholm, Sweden

**Keywords:** SWL, Extracorporeal shockwave lithotripsy, Indications, Treatment strategies, Precautions, Renal stones, Ureteral stones

## Abstract

It is well recognized that the popularity of extracorporeal shock wave lithotripsy (SWL), despite its non-invasive character, has decreased during recent years. This is partly explained by the technological achievements in endoscopy and urologists’ enthusiasm for such procedures. Another explanation is that many urologists have been insufficiently successful with SWL. The latter effect might to some extent be a result of the performance of the lithotripter used, but in too many cases, it is evident that the principles of how shock wave lithotripsy should be carried out are poorly applied. The purpose of this article is to emphasize some important aspects on how SWL best should be used. Based on decades of experience, it stands to reason that success with SWL does not come automatically and attention has to be paid to all details of this technique.

## Introduction

A careful review of the literature discloses that results of extracorporeal shockwave lithotripsy (SWL) vary considerably from one centre to another and from one operator to another. As a consequence of occasionally poor treatment results, we can notice that the popularity of SWL has decreased during the past decade. This development is to a large extent explained by the technical development of instruments for endoscopic procedures and increased skill in the application of these techniques, but this development is also a result of insufficient attention to the basic principles of how SWL should best be carried out.

As for every surgical procedure, it is important to apply proper indications for urinary tract stone removal with SWL. The important lesson learnt was that when the original Dornier HM3 lithotripter concept was abandoned and replaced by various later generation devices, the treatment, contrary to what was expected, became more difficult. It has been shown that strict control of a number of factors is fundamental for acceptable treatment results [1–3]. Neglecting these principles is certainly one of the most important factors that explain why during recent years, numerous SWL centres have failed to repeat the successful treatment results obtained with the HM3 equipment [4–12]. It is important to understand that in similarity with other surgical and medical procedures, SWL requires considerable skill and expertise by the operator [13–15].

To reach the therapeutic goal of efficient stone disintegration without increasing the risk of complications, it is necessary to make an appropriate selection of patients and, moreover, to pay careful attention to several important factors. For this purpose, it is important to obtain a careful medical history and to carry out the basic examination of the patient. Based on the details of the stone situation, anatomical features and possible risk factors, it is extremely important to inform the patient that repeated treatment sessions occasionally might be necessary and that repeated SWL should not be considered a failure but a consequence of the physics behind non-invasive stone disintegration [8, 9, 16]. With these pre-requisites, it is possible to carry out lithotripsy in a safe and harmonic way, also for patients with complicated stone situations. It is important not to fulfil any unrealistic goal by attempts to complete every stone treatment with only one session using excessive number of shockwaves and/or unnecessary high and risky energy levels.

Although experience has shown that the frequency of complications recorded after SWL is lower than that recorded with endoscopic and open surgical procedures, particular attention must be paid to the risk of developing renal subcapsular hematoma [17–22], other types of trauma to the kidney [23], injuries to surrounding organs, problems associated with infected urine or stones as well as consequences of urine flow obstruction caused by stones and fragments.

## Indications and contraindications

As already mentioned above, successful treatment cannot be anticipated without appropriate patient selection [1, 3, 23]. Moreover, the treatment has to be planned in detail according to the stone problem presented by the individual patient. The vast majority of patients has their diagnosis based on CT examination with or, most commonly, without contrast medium (NCCT). Aortic aneurysms [24, 25] and renal artery aneurysms can easily be identified to avoid shockwave direction to stones located nearby such abnormalities. The radiological examination also makes it possible to identify several other important morphological features. The stone size (longest diameter, stone surface area or stone volume), stone location, and stone density (Hounsfield units) should be determined. Such information is of importance for predicting roughly disintegration and need of repeated sessions or other necessary precautions.

There are few absolute contraindications for SWL. Pregnancy and *uncorrected* coagulation disorders are the most important ones [23–25]. These patients should not be treated with SWL despite occasional reports in the literature that it is possible. Another important pre-requisite for SWL success is that the fragments have a possibility to be eliminated from the renal collecting system. Patients with UPJ obstruction, ureteral strictures, or any other anatomical abnormality that will hinder fragment passage must be excluded [23–25]. The only exception to this rule are patients in whom combined SWL and PNL or combined SWL and percutaneous chemolysis [26] is planned. Skeletal deformities [25] might make SWL impossible. This might, for instance, be the case for patients with severe scoliosis, but if a favourable shockwave path can be established, such patients should not be excluded. SWL may be the only treatment alternative, because respiratory problems might make anaesthesia risky or even impossible. For patients with complex anatomy, it is of great help to test positioning in the lithotripter before definite decision for SWL.

Particular care with cautious SWL is strongly recommended for patients with specific risk factors. The most important of these conditions is summarized in Table 1. It is particularly important to avoid overtreatment of these patients, both in terms of shockwave energy and number of shockwaves. The rule is that it always is better to re-treat than to expose the patient to unnecessary risk of bleeding or tissue trauma.

**Table 1:** Patients with increased risk of SWL complications or for whom SWL otherwise should be carried out with great care

Hypertension
Borderline blood pressure (~ 140/90 mmHg)
Patients treated for hypertension
Cardiovascular disease
Diabetes mellitus
Reduced renal function
Children

It is generally accepted that patients with a large stone burden are poor candidates for SWL [25, 27]. Patients with staghorn stones should not be considered for SWL treatment and the same recommendation is applicable for all patients with large volume non-staghorn stones. One exception to this rule are those patients in whom stone disintegration with SWL can be combined with percutaneous surgery and/or chemolysis [26].

The upper stone size over which preference is given to invasive procedures such as PNL and RIRS varies slightly from one centre to another. It is, however, generally considered that stones in the kidneys with maximum diameters up to 20 mm (stone surface area of in averages approximately 200–225 mm^2^) are suitable for SWL [25]. That size limit is occasionally expanded to 20–30 mm (corresponding to stone surface areas of approximately 225–450 mm^2^) for stones with low hardness [28].

It is also commonly not recommended to use SWL for lower calyx stones with diameters exceeding 15 mm (stone surface area of 115–180 mm^2^). Some authors have suggested an upper size limit as low as 10 mm (approx. 50–80 mm^2^) [28, 29]. This recommendation is based on the fact that most residual fragments are found in the lower calyx. It is of note, however, that a majority of renal stones initially are located in that part of the kidney. It also is necessary to emphasize that residual fragments in the lower calyx not only come from stones initially located there, but also from stones disintegrated in other parts of the renal pelvis and calyces. It is not known whether it is the size of the stone that is the limiting factor for clearance of the calyx or if it is the long-term dilatation by large stones that negatively affect contractility of the calyx muscles.

If stones with diameters exceeding 20 mm (> 200–225 mm^2^) are selected for SWL, it is wise *to exclude* those composed of brushite and cystine (compact type) and probably also COM because of their well-recognized SWL resistance [28, 30, 31].

The need of repeated SWL can never be absolutely excluded and it is always necessary to be aware of the need of repeated treatment sessions when large and hard (SWL resistant) stones or bigger lower calyx stones are planned for SWL. If repeated sessions cannot be accepted, endoscopic procedures preferably should be used [25]. Decisions to treat large and hard stones with SWL need careful consideration of the patient’s individual stone situation, risk factors, and informed personal preferences.

**Fig. 1:**
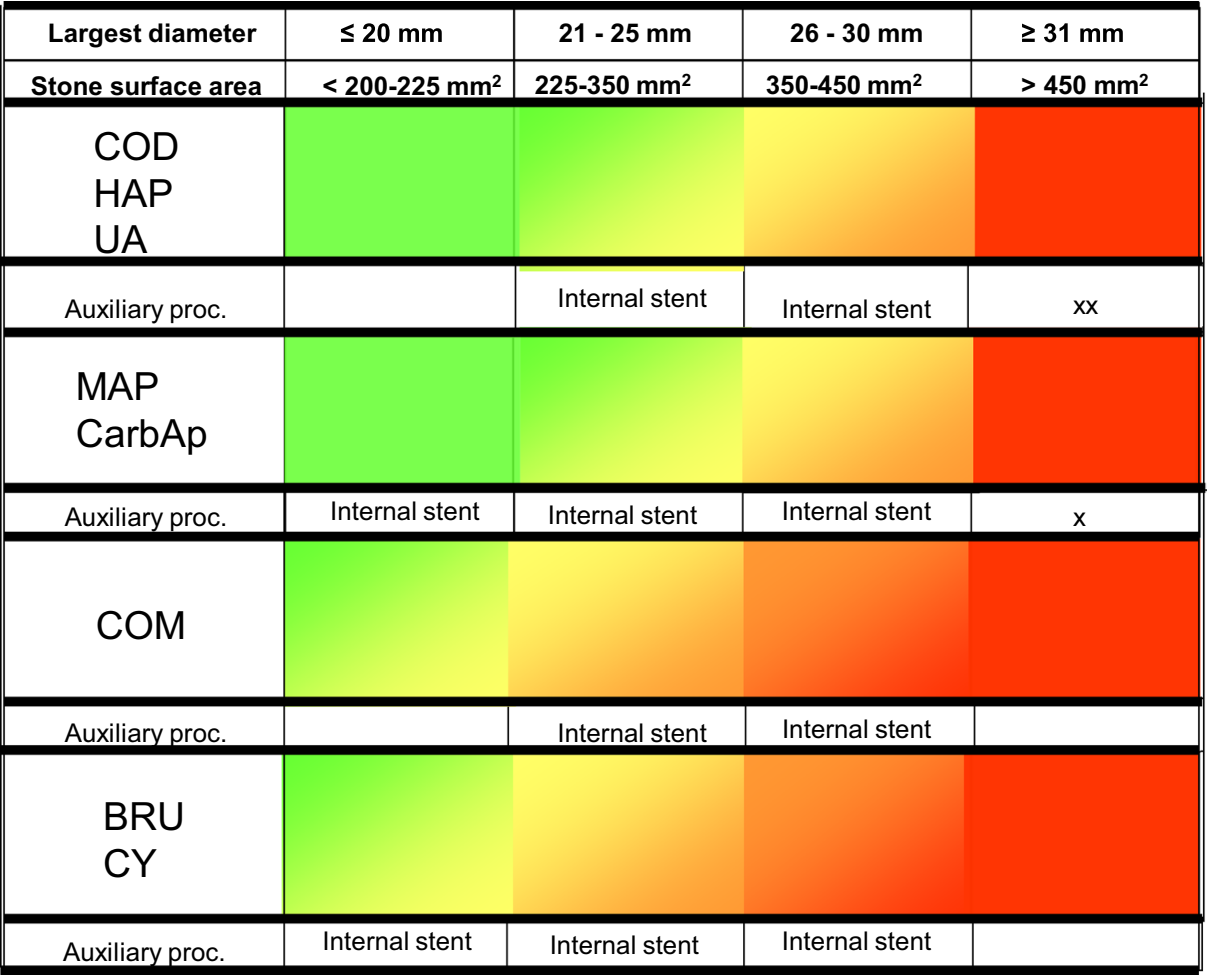
Overview of indications and estimated clinical success with SWL for stones in the kidney. *Green* indicates that the stone situation is ideal for SWL and *red* that it is not. *Yellow* means that the stone can be successfully treated and provided that repeated treatment sessions can be accepted. For stone situations shown in *orange* colour, SWL is not recommended as primary treatment but might be considered as a rescue method when other alternatives have been excluded. It should be noted that there are several other factors that also need to be taken into account such as anatomical features of the renal collecting system, body habitus, and various concomitant health problems. Stone surface area (SA) was calculated from the length × (length × 0.65) × *π* × 0.25

**Fig. 2:**
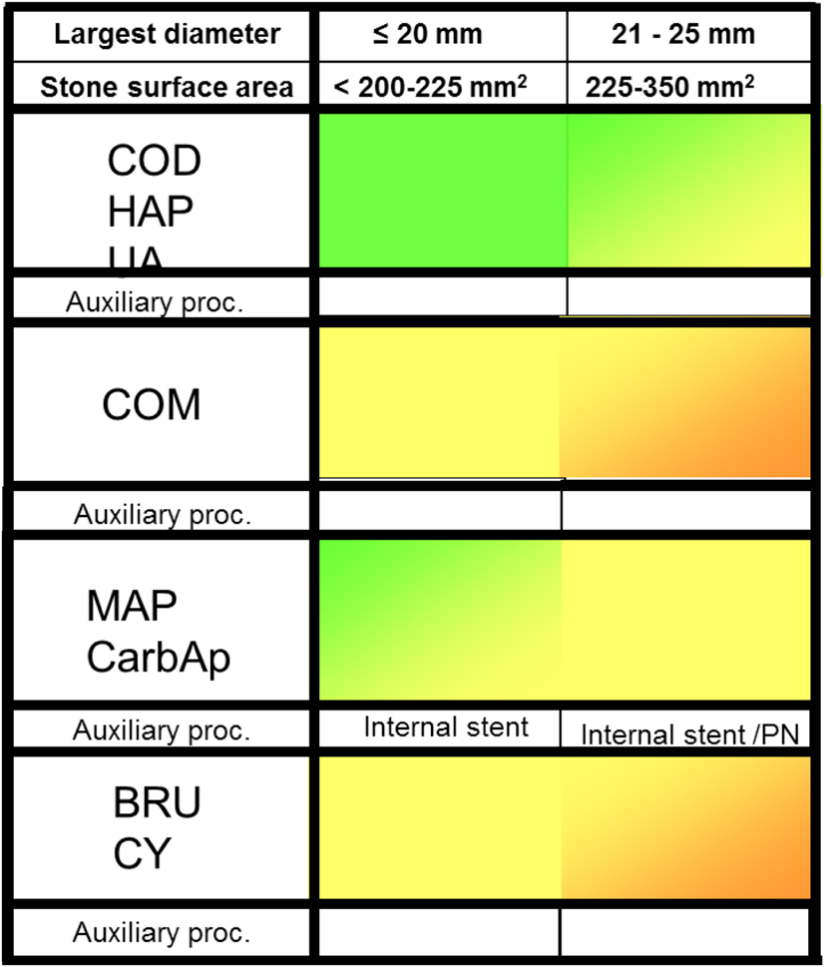
Overview of indications and estimated clinical success with SWL for stones in the ureter. *Green* indicates that the stone situation is ideal for SWL and *yellow* that the stone situation can be successfully treated and provided that repeated sessions can be accepted. It should be noted that there are several other factors that also need to be taken into account such as anatomical features of the renal collecting system, body habitus, and various concomitant health problems. Stone surface area (SA) was calculated from the length × (length × 0.65) × *π* × 0.25

A simplified overview of indications, considerations, and decisions for SWL monotherapy for treating patients with stones with different compositions in the kidneys and ureters is shown in Figs. 1 and 2. The scale goes from green to red. *Green* means that the stones comprise suitable indications for SWL and that stone problems shown in *red* are unsuitable for SWL. The *yellow* colour shows that the stones can be treated with SWL and provided that repeated treatment sessions can be accepted. *Orange* stone situations are generally no indication for primary treatment with SWL, but when other treatment alternatives have been excluded for some reason, SWL might be used as rescue treatment.

Reports on the geometrical anatomy of the lower calyx system are contradictory and the results inconsistent [32–38]. The bottom line is that fragment clearance generally is facilitated from kidneys with short infundibulum, large calyx width, short height from the stone position to the lower calyx lip, and obtuse angle between the stone bearing calyx and the outflow from the pelvis or the vertical axis [34]. A combined estimate comprising the calyx height and the calyx length can be used to summarize the lower calyx anatomy. That estimate also includes the cosine of the angle between the calyx direction and the vertical axis [35]. The suggested discriminating variables are not easily measured on CT examinations, and prediction of stone clearance from these variables is difficult even when contrast medium has been administered.

Hardness of stones is reflected in terms of Hounsfield units (HU) and approximate limits for stones of different compositions have been published [39–42]. If a previous stone analysis is available, it is of course most valuable information, because comparison of stone composition and treatment efforts has disclosed that the most shockwave resistant stones are composed of COM, brushite, or cystine [30, 31]. The latter stone is resistant to shockwaves because of its entirely organic structure. Clinical and experimental studies have shown two different types of cystine stones: one with a brittle structure relatively easy to disintegrate and one with a solid and more resistant morphology [43]. It is important to know, however, that although the mentioned stones are more difficult to disintegrate with SWL than those composed of calcium oxalate dihydrate, hydroxyapatite, carbonate apatite, magnesium ammonium phosphate, and uric acid, they can nevertheless be disintegrated with SWL provided energy levels are appropriately chosen and repeated sessions are accepted [1, 3].

**Fig. 3:**
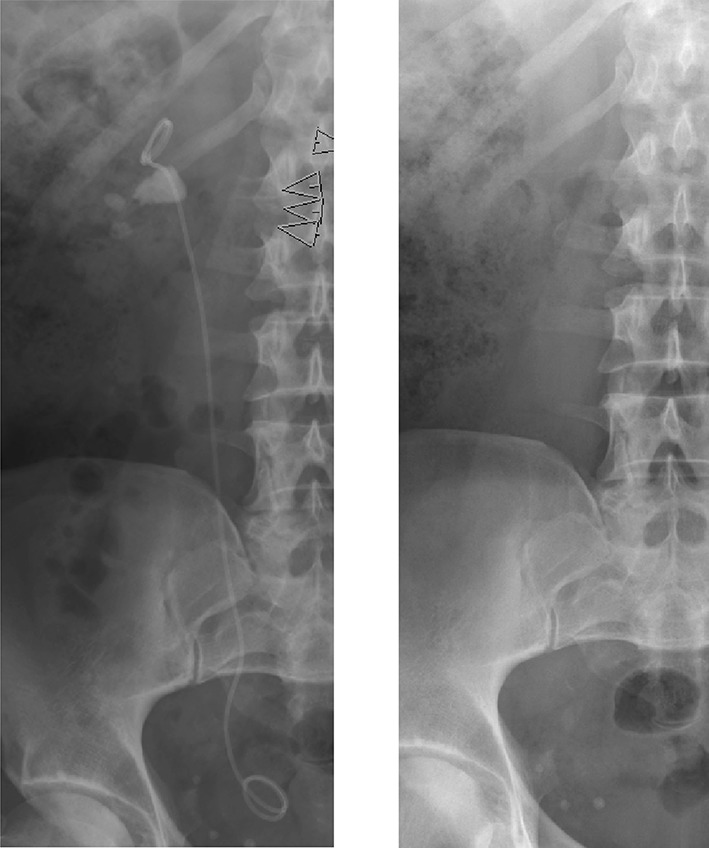
Large stone with an internal stent to ovoid steinstrasse. Before SWL (**a**) and after (**b**)

As shown in Fig. 2, stones at all levels of the ureter are excellent indications for SWL [44]. In some recommendations and guidelines, an upper stone diameter limit of 10 mm (~ 50 mm^2^) has been suggested for ureteral stones [25], but it has been repeatedly demonstrated that also larger stones advantageously can be treated with good results. The rule to insert an internal stent before treatment of large stones in the kidney (> 20 mm; >200 mm) is usually not applicable to ureteral stones, unless there is pronounced obstruction [45]. There are several observations indicating that stone disintegration in the ureter can be negatively influenced by the presence of a stent, and in many patients, it accordingly can be wise to consider stent removal before SWL. For large stones in the *kidney*, it is recommended to insert an internal stent (Fig. 3).

It is well recognized that successful disintegration of stones in obese patients is difficult. This problem is to some extent explained by the limited penetration depth of many lithotripters. With too short penetration depth, the stones cannot be appropriately placed in the shockwave focus. There is also a considerable loss of energy in both abdominal fat and muscle tissue during the long shockwave passage [46, 47]. It has been shown that in patients with a skin-to-stone distance (SSD) of more than 100–110 mm and a stone density exceeding 900–1000 HU, the likelihood of stone disintegration is significantly reduced [48–52]. The large distance to the stone in obese and large patients can sometimes successfully be overcome using an extended shockwave path: “blast path” technique [53, 54]. It might in these patients also be necessary to use higher energy levels than those commonly used for disintegration of stones in non-obese patients [23].

## Measures aiming at reduced risk of haemorrhage and renal injuries

**Fig. 4:**
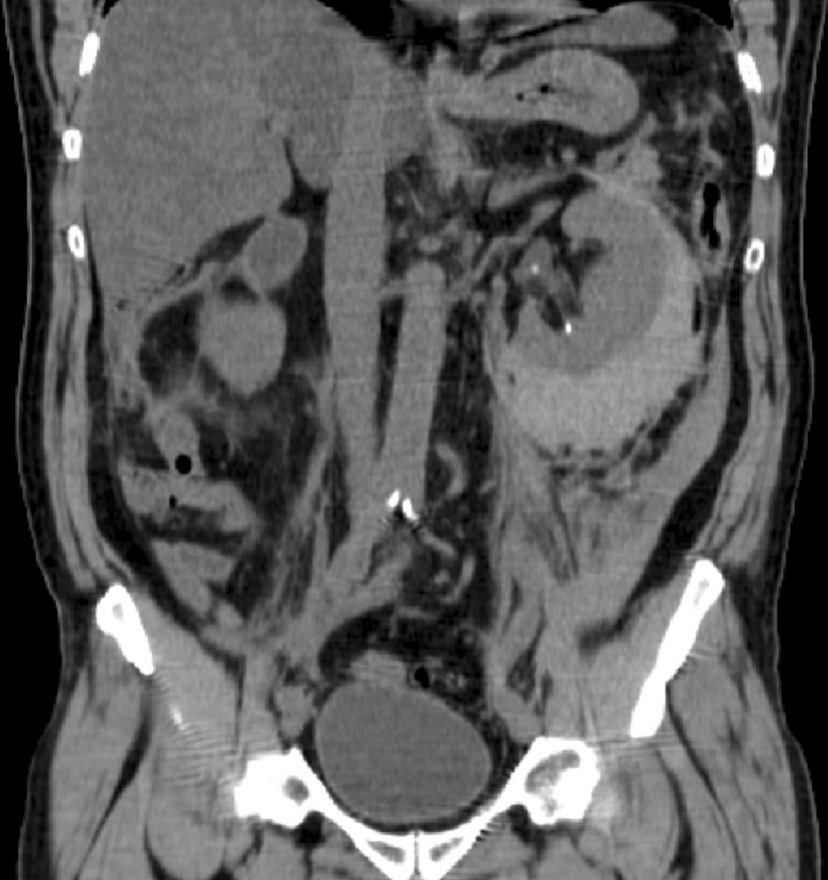
Patient with subcapsular hematoma on the left side after SWL

One of the most serious and feared complications of SWL is the development of renal subcapsular hematomas (Fig. 4). With this complication, that in most series fortunately does not occur more frequently than in approximately 1%, the blood loss is considerable and the effects on the renal function deleterious. Subcapsular hematoma is a consequence of rupture of large vessels in the renal capsule. There is an increased risk for this complication in patients with high blood pressure [55]. It is, therefore, compulsory to measure the blood pressure in every patient for whom SWL is planned and also to measure the pressure intermittently during the treatment. When hypertension is recorded, the pressure should be normalized before shockwaves can be directed towards stones in the kidney. The same restriction is necessary for all those stone treatments associated with risk of hitting the renal tissue with shockwaves. This might happen, because the shockwave focus is close to the kidney or respiratory movements lead to the risk of bringing renal tissue into focus [56]. It is necessary to be aware of this problem when treating proximal ureteral stones. Shockwave disintegration of stones in the mid and distal ureter is, however, not a problem in this regard, but in all other patients, antihypertensive treatment should be given to establish a normal blood pressure before SWL.

It is important to realize that patients with a history of hypertension (albeit normotensive at the time of treatment), diabetes mellitus, and generalized vascular calcifications with fragile vasculature might have increased risk of developing renal hematoma and, therefore, must be treated with great care.

Macroscopic hematuria is often reported as a complication to SWL. The details of this observation are seldom described, but a limited period of macroscopic hematuria appears routinely relatively early during SWL. This bleeding is a consequence of injuries to the urothelium caused by stone fragments tearing the mucosa [1, 3]. Long-term experience has shown that without an episode of macroscopic hematuria, stone disintegration has probably not occurred. The authors have made this observation in thousands of SWL treatments carried out with high diuresis and bladder catheter. If SWL is not accompanied by hematuria, the treatment variables should be adjusted or the stone position in the lithotripter reconsidered. Warning for treating stones located adjacent to the renal capsule, there is a significantly increased risk of vascular injuries, and in such cases, the decision to proceed with SWL should be reconsidered.

Patients with anti-platelet treatment of any kind are at significant risk of bleeding complications and treatment should not be carried out until this risk has been eliminated [55, 57, 58]. For patients treated with acetylsalicylate preparations (ASA, for instance, aspirin; however, there are today numerous pharmacological agents containing ASA), the medication should be deferred to allow for new thrombocytes to form. Various recommendations are given in the literature, and the current advice is to withhold the treatment for a period of 7 days before SWL [58–60]. Zanetti and co-workers stopped ASA for 8 days and the EAU guidelines previously recommended 10 days [61], corresponding to the time required for renewal of thrombocytes. The authors of this review have maintained the 10-day rule. Irrespective of the duration of the treatment pause, it is a good habit to measure the bleeding time in these patients just before SWL to make sure that no tablet accidentally has been ingested during the therapy-free period. For new anti-platelet agents, consultations with pharmacological experts might be necessary.

Although diclofenac is associated with a theoretically increased risk of bleeding complications, clinical experience has shown that such treatment does not increase the risk of bleeding and there are accordingly no recommendations to defer diclofenac. In fact, many patients are given diclofenac routinely, both before and after SWL [3, 62].

Patients on anticoagulation treatment with warfarin or other similar agents need special care and internal medicine consultation is suggested to make sure that this form of anticoagulation therapy temporarily can be stopped [60]. The same advice is applicable for all new anticoagulation agents. If this form of treatment cannot be withheld, the patient should be treated endoscopically with URS or RIRS [60]. Endoscopy has in fact been recommended as the best alternative for these high-risk patients. For patients in whom warfarin administration temporarily can be discontinued, the latest dose should not be taken later than 4–5 days before the planned SWL session [60]. INR needs to be measured on the day of treatment and should not be higher than 1.2. During the period without warfarin, bridging therapy should be given with low-molecular heparin (Fragmin®; dalteparin sodium). The first dose should be administered subcutaneously on the day of treatment, but after the SWL session! The daily dose of Fragmin should never exceed 2500–5000 units. Re-start of warfarin treatment is determined by the further planning of stone removing procedures or according to internal medicine recommendations, but warfarin should never be reinstituted earlier than 48–72 h after SWL [60]. Patients with other bleeding disorders need special considerations and preparation by coagulation experts, but when the abnormality is temporarily corrected, gentle SWL is possible in most cases.

Several studies have shown that when shockwaves are administered at a low rate, the injuries to the renal parenchyma are much less pronounced than when a high frequency SWL is used [63–71]. This observation is particularly important to consider when treating patients with increased risk of bleeding or renal injury. With slow-rate shockwave administration, it is possible to avoid or decrease the negative effects of cavitation bubbles that remain in the tissues at the time of the next shockwave [71]. In most investigations, a comparison has been made between the frequencies 120 and 60, but whether a frequency of 60 is better than 90 has not been established. On the other hand, it has been shown that with a frequency of 30 even less undesirable tissue effects were recorded [23, 72]. The latter frequency is, however, not easily applied in most lithotripters, but there are definitely arguments in favour of a frequency of 60 [63–67, 70]. A slow rate of shockwave delivery is recommended for patients with the specific risk factors, as listed in Table 1. The technical details of SWL are further discussed below.

SWL of stones in the kidney always results in more or less pronounced contusions and it is, therefore, wise not to repeat the treatment until sufficient recovery has been established [73]. The recommended interval between successive sessions, directed towards the kidney, is 10–14 days, at least with the high energy densities in modern lithotripters.

Several methods have been described to counteract tissue injuries by elimination of free radicals and decreasing oxidative stress. It has been shown that calcium channel blocking agents as well as different anti-oxidants and free radical scavengers favourably decreased injurious SWL effects on the renal tissue. Verapamil, nifedipin, allopurinol, vitamin C, vitamin E, selenium, and melatonin have been used successfully either clinically or experimentally [74–79]. Although the routine use of such agents is uncommon, it might be useful to keep this option in mind and pre-treat patients with reduced renal function. In addition, for patients in whom it is likely that repeated sessions might be necessary, such measures can be helpful to counteract or decrease renal injuries.

Whether hypertension is a late complication of SWL has remained a matter of debate over the years [80]. Although some studies have indicated such a relationship, there are now several studies demonstrating that SWL does not result in hypertension [81, 82]. The difficulty to appropriately interpret results in long-term follow-up studies is that there is a correlation between hypertension and stone disease. A similar relationship between diabetes mellitus and stone formation is also well recognized, but development of diabetes as a consequence of SWL can also with a great degree of certainty be excluded based on clinical as well as animal studies [82, 83].

Occasional organ-confined complications such as acute pancreatitis and damage to the pancreas, spleen, liver lungs, and intestinal have been reported. Fortunately, all these complications are extremely rare.

One important question is if SWL of distal ureter stones might have negative effects on the gonads and reproduction. Fortunately, both animal experiments and clinical experience have shown that SWL close to ovaries [84] and testicles [85] can be carried out without late risk of injuries. All observed tissue effects in these organs have been transient. There are few studies that have addressed the danger of radiation to the gonads during fluoroscopy, but the clinical effects during the past 30 years have not indicated any deleterious effect.

## How to avoid infection complications?

The second major risk of complications is associated with bacteria that are present in urine and/or the stone and released or disseminated during stone disintegration. With subsequent obstruction to the urine flow, life threatening septicaemia and septic shock can emerge. It is of fundamental importance to identify infection risk factors before proceeding to SWL. A test for bacteriuria is mandatory for all patients. When positive, a urine culture should be added for subsequent appropriate treatment with antibiotics. In addition, when bacteriuria has not been demonstrated, it is necessary to treat the patient with antibiotics when there is a medical history of urinary tract infections or when the stone morphology indicates that infection might have played a role in stone formation. In patients with a positive urine culture and sensitivity pattern available before the planned SWL, pre-treatment with appropriate antibiotics should always be made. When the demonstration or suspicion of bacteria is identified on the day of SWL and in the absence of results from urine culture, empirically intravenous administration of broad-spectrum antibiotics such as an aminoglycoside or ceftazidim 1 h before the treatment has proven clinically useful [3, 86]. However, it is also a good routine to insert an internal stent in medically weak patients to avoid the risk of ureteral obstruction, irrespective of the size of the stone(s) [3]. All patients with percutaneous nephrostomy tubes have bacteriuria and they should be given intravenous antibiotics before the SWL session, also in cases of a negative test for bacteriuria.

Patients who are planned for SWL after an episode of serious urinary tract infection with or without septicaemia need both decompression of the renal collecting system and appropriate treatment with antibiotics. It is generally recommended to wait with SWL for a period of at least 2 weeks after successful infection treatment.

## How to deal with obstruction following SWL?

**Fig. 5:**
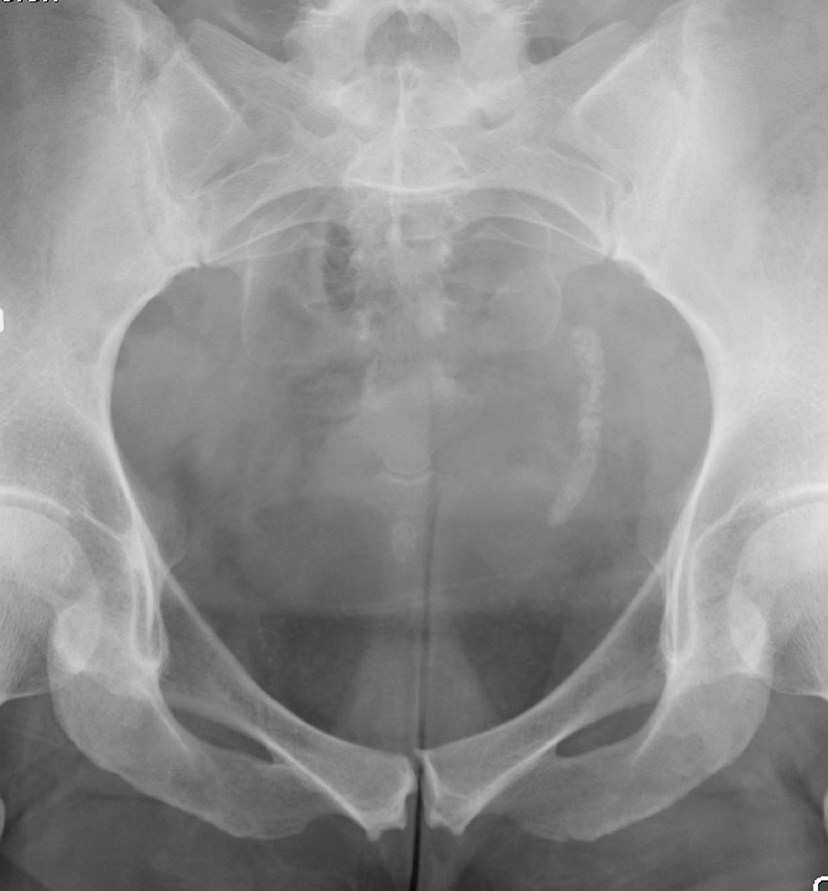
This is a typical steinstrasse in the distal left ureter. Columns of fragments are seen after all stone disintegrations, but steinstrasse is the clinically important condition in which the fragment accumulation is associated with obstruction to the urine flow

Disintegration of stones should always result in fragments or small stones aimed to be eliminated with urine through the ureter. Accumulation of impacted fragments in the ureter causes obstruction (Fig. 5). This condition is termed “Steinstrasse”, which increases with the initial stone volume. Both isolated stone fragments and “steinstrasse” can in most cases advantageously be treated and eliminated with repeated SWL.

**Fig. 6:**
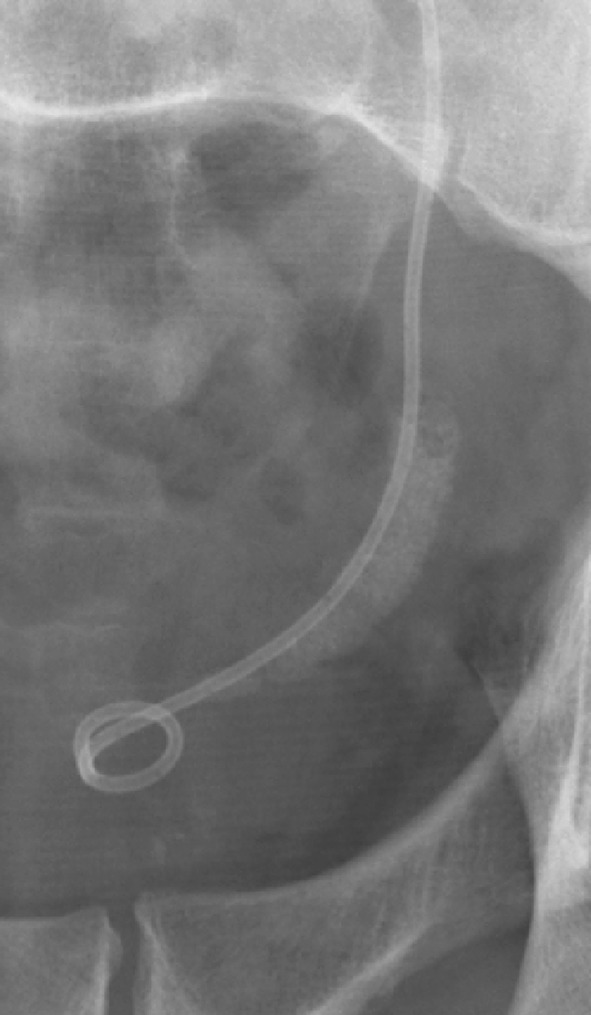
Fragment accumulation alongside an internal stent. This stent was gently passed through the fragment mass before repeated SWL

To avoid the problem of obstruction caused by large fragment volumes, it is helpful to insert an internal stent before stone disintegration [61]. This procedure is in most cases recommended for stones with a diameter exceeding 20 mm or stone surface area above 200–300 mm^2^ (Fig. 3). Despite reports in the literature that stenting is not necessary, it is the authors’ opinion that many problems can be avoided with such precaution. Insertion of an internal stent can in most patients conveniently be carried out under local anaesthesia, with or without administration of low-dose analgesics and sedatives. Only very exceptionally will ureteroscopy be necessary to remove a “Steinstrasse”. Most cases can be dealt successfully with SWL, with or without insertion of a ureteral stent (Fig. 6). Childrens’ ureters have a much better transport capacity than those of adults and stenting can, therefore, be omitted in most children also before SWL of larger stones [87]. Stenting can also be recommended for weak persons in whom every kind of obstruction might be deleterious.

When ureteral obstruction is associated with impaired renal function and/or infection, it is mandatory to insert either an internal stent or a nephrostomy tube before any other procedure. In case of infection, appropriate treatment with antibiotics should be started immediately.

## Pacemakers and defibrillators

The general rule is that patients who have pacemakers or defibrillators can be treated with SWL. Few such devices might, however, be disturbed or inactivated by the electromagnetic field of shockwaves. Before treating such patients, it is important to consult the responsible cardiologist. Detailed recommendations on how to deal with this problem can be found in the literature [88, 89].

## Technical aspects on how to carry out the shockwave procedure

The fundamental pre-requisite for successful SWL is that the shockwave can pass into the body and hit the stone with minimal loss of energy. When the original Dornier HM3 lithotripter was used, the shockwaves were generated in the same water compartment as the patient. In modern lithotripters, there is a gap between the shockwave source (therapy head) and the body that has to be bridged. This interface is an obstacle that is overcome by application of a transmission medium: usually ultrasound gel, silicon oil, or occasionally some other medium. It is extremely important to avoid the simultaneous application of air bubbles when the transmission medium is applied on top of the therapy head. In addition, the presence of only relatively small volumes of the air in the transmission gap will attenuate the shockwave power and in the worst case completely extinguish the energy of the shockwave. Therefore, meticulous care is necessary when the transmission medium is applied to get optimal coupling. Pumping devices and bottles containing the air should never be used and shaking of any such vessel is strictly forbidden. It is recommended to apply large quantities of the transmission medium and carefully spread it out over the surface of the therapy head. It is generally not possible to have control of the coupling interface and to determine whether air bubbles are present or not when the therapy head is in position. Devices using video cameras have been developed to check if the transmission zone is free of air bubbles and the coupling is optimal [1, 3, 90, 91]. This equipment recently has been incorporated in the therapy head of some lithotripters with obvious advantage.

The next steps in the SWL procedure are identification of the target and optimal positioning of the patient [1, 3, 92]. Fluoroscopy is most commonly used [14], but ultrasound might also be used. The latter technique is used for identification of the radiolucent uric acid stones as an alternative to contrast medium. The current recommendation for fluoroscopy is to use the pulsed technique with the aim of reducing the X-ray load. This method is, however, associated with the difficulties to adjust for respiratory movements, and at least during parts of the procedure, it is advisable to use continuous fluoroscopy. It has been demonstrated that the success rate of SWL correlates with the fluoroscopy time [93]. It is, however, very important to keep the radiation load as low as possible and the collimators should be used early during the SWL treatment to maximally reduce the radiation field. Neglecting this important rule is widespread with unnecessary large radiation doses! As an example, the radiation dose measured with wide open aperture was approximately 20 times higher than that recorded following collimation of the aperture to a square of 7 × 7 cm! This is a problem both for the patients and the operating staff. It is likely that improvements in ultrasound technology will result in the future expanded use of stone localization and positioning without fluoroscopy.

For treatment of stones in the kidney or proximal ureter, the hit rate of the shockwaves is occasionally low, as a result of the patient’s respiratory movements [1, 3, 56, 93]. The distance over which the stone moves sometimes can be substantial. The basic rule that should be applied is to place the stone in focus during the expiratory phase. Another way to counteract this problem is to apply a belt or a plate across the upper part of the abdomen [1, 3, 94, 95]. In addition to a better hit rate, this trick makes sure that the patient has optimal contact with the shockwave source. Patients who feel uncomfortable otherwise often elevate the body from the therapy head. Attempts with respiratory triggering have been less successful because of irregular respiratory movements during SWL and the hit rate nevertheless remains low. High frequency ventilation might be used to increase the hit rate, but that technique requires general anaesthesia, a factor that will offset the advantage of anaesthesia free treatment.

**Fig. 7:**
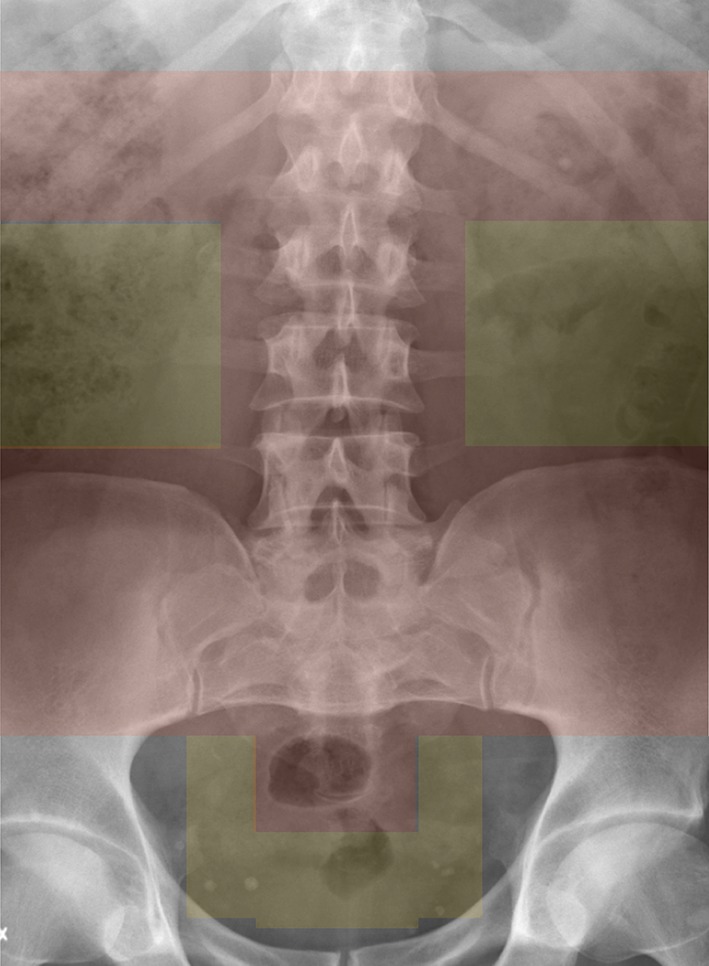
Different areas are indicated for which it is necessary to consider if shockwaves best are administered from the back (yellow) or trans-abdominally (red). The decision has to be based on the patient’s anatomy as well as on which type of lithotripter that is used

With the stone in focus, it is important to make sure that no skeletal structures interfere with the shockwave path [1, 3]. With delivery of shockwaves from the back of the patient, such interference might be caused by transverse processes, ribs, and different parts of the sacroiliac-bone massive and pelvic skeleton. How to manage this problem and allow for free shockwave path in the body differs from one lithotripter to another and can also vary between individuals. Figure 7 indicates different zones in this regard. Green fields indicate when SWL usually can be carried out with shockwaves directed from the back and red zones when transabdominal administration might be necessary or worthwhile to consider. Stones located in the angle between the spine and pelvic brim constitute a common problem, irrespective of lithotripter, because in this area, the skeleton has a considerable uptake of energy and a similar problem is typical also for stones located immediately below the sacroiliac joint. Adjustment of the patient’s position in most situations can solve the problem and still enable administration of shockwaves from behind. If this is impossible or difficult, it is recommended to administer shockwaves from the abdominal side. Such an approach is always necessary for stones located in the mid ureter and sometimes for selected patients with stones in the distal ureter [44]. In several reports, superior results have been presented with trans-gluteal shockwave administration, usually with the patient in supine position [44, 97].

**Fig. 8:**
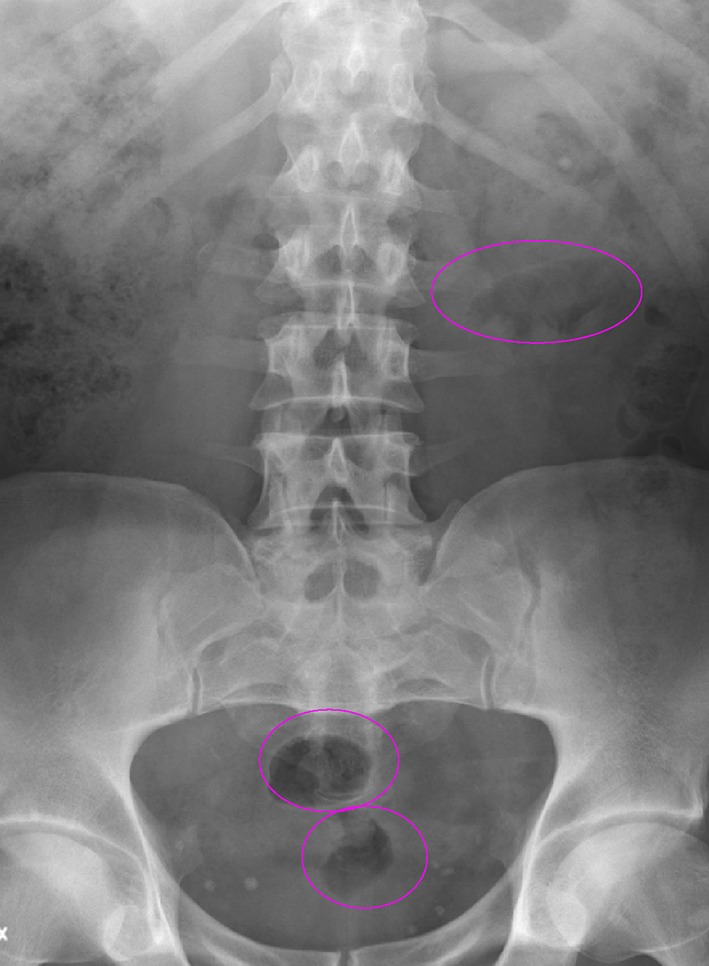
Problem with intestinal gas needs to be dealt with when it is necessary to administer shockwaves trans-abdominally

The problem with transabdominal shockwave administration is interference with intestinal gas. When gas is found in the shockwave path, no treatment should be carried out, because most or all energy will be extinguished and the risk of causing trauma to the intestinal wall is increased. Gas in the left colon (Fig. 8) can usually be eliminated with an appropriate enema [3]. It might occasionally be successful to move or eliminate intestinal gas with massage. For all patients in whom shockwave administration from the abdominal side might be expected or cannot be excluded, pre-treatment with dimeticon is an option [3]. Pre-treatment with laxative is not necessary, and in a non-published comparison between patients given laxative or not before urography, there was no advantage with the first regimen, and if any difference was observed, it was worse after intestinal preparation.

Animal experiments and clinical experience have shown that the risk of tissue injuries with ensuing bleeding can be reduced by starting SWL treatment with a series of shockwaves at low energy level, for instance, 100 shockwaves [64, 98–103]. A favourable vasoconstriction can be achieved when this initial treatment is followed by a pause of few minutes [104]. Recent experience has shown, however, that vasoconstriction probably can be obtained also without such a pause but with a longer treatment with low power shockwaves. In the further course, it is recommended to stepwise increase the power. This treatment modality is termed *ramping* and has several advantages. First, it is easier for the awaken patient to adapt to the treatment. Second, ramping facilitates conclusion at which level stone disintegration starts and overtreatment in terms of energy can be avoided. Moreover, several studies have shown that ramping results in better disintegration than when the power rapidly is increased to high levels or when a high power level constantly is used during the treatment. It is, furthermore, important to know that high energy levels result in large fragments and low energy levels in small fragments. Nevertheless, it is necessary to exceed a certain energy threshold to overcome the attractive forces between the stone crystals.

Excessive administration of shockwaves during one session might result in contusion and other tissue injuries [104]. It is important not to over-treat the patient neither in terms of shock wave number nor shockwave power [23, 105]. The most commonly recorded upper limit of the number of shockwaves in the literature is in the range of 2000–4000. This level needs to be adjusted relative to the total energy given. It is necessary to closely follow stone disintegration. Treatment should not be continued when sufficient disintegration has been confirmed. Moreover, when it is less likely that any further disintegration will occur unless fragments surrounding the stone are eliminated, treatment should be terminated. Standard routine programmes that always use the same upper number of shockwaves should probably be modified when a low shockwave frequency is used because of better disintegration. For all patients with specific risk factors (Table 1), a low number of shockwaves and low energy levels should be used and the same principles should be applied when treating children.

It is not possible to give strict recommendations on the total number of shockwaves that should be administered. Stone size and hardness as well as the patient’s body features are important determinants [20, 46, 48, 53]. Careful observations of the course of the SWL treatment should support the decision for termination of the treatment. Fragments covering the stone will attenuate the shockwave power. If a stone has not been adequately disintegrated after 3000–4000 shockwaves, it is almost always better to repeat the treatment at a later occasion than to over-treat the patient. Clinical as well as experimental studies have indicated that a shockwave frequency of 60 (1 Hz) is better than 90 (1.5 Hz), but for risk patients and children, our recommendation is to always use a frequency of 60.

## Treatment of pain during SWL

One important pre-requisite for successful SWL treatment is to give the patient sufficient pain relief [1, 3]. For the vast majority of patients, the whole treatment procedure can be completed with analgesics and sedation only. General anaesthesia should usually be reserved for children. There are different methods for anaesthesia–free SWL described in the literature [6–14]. It is the authors’ experience that small intermittent doses of alfenatyl and propofol are both safe and very efficient [3]. Great care must be taken to make sure that the treatment is dictated by the course the stone fragmentation and not by the patient’s reaction. The treatment will be unsuccessful if the shockwave energy needs to be maintained at an insufficient level, because the patient has inadequate pain relief. During the procedure, the patient should have supply of oxygen, recording of pO_2_ and ECG and repeated measurement of blood pressure.

Is high diuresis during SWL beneficial? This issue has remained a matter of debate. It was shown in a comparative study of SWL for ureteral stones that the outcome was similar for patients with and without high diuresis [106]. For stones in the kidney, a high urine flow might, at least theoretically, be favourable. One advantage with high diuresis and bladder catheter is that this arrangement enables detection of early hematuria which is a reliable sign of stone disintegration [3]. This observation might be helpful when difficulties in stone identification and positioning can be anticipated. It is also useful for teaching new operators how to manage SWL. According to our experience, the slightly more invasive character of the treatment by insertion of a bladder catheter and intravenous fluid infusion is surprisingly well tolerated by the patients. To achieve high diuresis, high-pressure infusion of 1000 ml Ringer solution during the treatment together with 20 mg of furosemide has been rewarding.

As a result of administration of analgesics and also of the SWL treatment itself, the heart frequency might occasionally be low. This problem is in most cases best corrected by i.v. administration of 0.5 mg of atropine.

Infusion of contrast medium is sometimes helpful for stone localization. The best conditions are obtained with retrograde contrast via a ureteral catheter or by antegrade infusion percutaneously in those patients who have a nephrostomy tube in place. Intravenous contrast injection is only occasionally helpful, because the filling of the collecting system is only of very short duration. Intravenous injection of contrast medium does not make sense for patients who have been given diuretics. Before injection of contrast medium, it is necessary to make sure that there are no contraindications such as reduced renal function, allergy, or treatment with methformin.

## Post-SWL treatment and follow-up

SWL always should be terminated when the disintegration is considered optimal or when it is likely that further administration of shockwaves will be without effect. Several pharmacological approaches have been suggested for improving passage of fragments and for making fragment passage as comfortable as possible.

Inasmuch as passage of fragments will cause episodes of pain, administration of diclofenac suppositories twice a day during the first 5–7 days after SWL has proven useful both for alleviating pain and to reduce the number of emergency visits [62].

Although different treatment results have been reported in the literature, numerous studies have shown that fragment passage both from the ureter and kidney can be facilitated by administration of α-receptor antagonists or calcium channel blocking agents [45, 107–110]. This kind of treatment apparently has capacity to reduce pain experience.

Administration of diclofenac 50 mg × 2 during 5–7 days, an α-receptor antagonist 4 mg × 1 up to 4 weeks after the treatment and furosemide 40 mg × 2 (together high fluid intake) during 5 days might be of value.

The principles of follow-up depend on how the immediate outcome of the SWL treatment is judged. In the literature, the majority of results of SWL for stones in the kidney are reported after a follow-up period of 2–3 months. In uncomplicated cases, it is possible to carry out the imaging control even later. Earlier follow-up is recommended when it is assumed that the disintegration is incomplete and it is likely that repeated SWL will be necessary. When stones in the kidney have been treated, there is generally no reason to proceed with follow-up examination earlier than 10–14 days after treatment. The exception to that recommendation is when symptoms raise suspicion of urine obstruction or other complication. Ultrasound control after 24 h has been reported helpful for determining stone disintegration and excluding kidney trauma.

There is no definite recommendation on the shortest interval between successive SWL sessions. It is the authors’ opinion that for treatment of stones in the kidney, the interval between repeated SWL sessions usually should not be shorter than 10 days. This rule is, however, not applicable for treatment of stones in the ureter and such treatments can be repeated after only a few days, provided that the renal tissue will remain outside the focal area. Some authors routinely carry out a KUB examination on the same day as the treatment or on the following day. In our experience, such an early follow-up generally adds very little information to that already obtained with fluoroscopy during the SWL procedure.

Exactly, how the follow-up imaging should be carried out remains a matter of debate. Unfortunately, uncritical overuse of CT examinations results in extensive exposure of the patients to radiation. For radiopaque stones, it is in most cases sufficient with standard plain film examinations (KUB) [111]. In selected cases, KUB advantageously can be combined with ultrasound imaging. In asymptomatic patients, urography or CT with contrast is seldom necessary and high-quality KUB is usually sufficient to demonstrate absence of stone material in the ureter. In the early follow-up, it should be noted, however, that the only early indication of “Steinstrasse” might be a slight elevation of the body temperature without pain and with only minor discomfort. Early decompression of the renal collecting system with an internal stent or a percutaneous nephrostomy tube is the appropriate management of that condition [45]. Moreover, in patients who present with pain of unexpected severity shortly after the SWL: always suspect subcapsular haematoma and refer the patient to NCCT examination and haemoglobin analysis!

Additional instrumentation or repeated SWL (or alternative endourological procedure) might be necessary for the appropriate management of residuals. Residual fragments or stones composed of uric acid are best treated with oral chemolysis by administration of alkali [26, 112]. To avoid early recurrent stone formation, it is particularly important to clear the kidney from infection and cystine stone residuals. Thereby, it might be necessary to use RIRS or PCNL. In selected patients, unfit for surgery, and with a percutaneous nephrostomy in place, chemolysis is an option for dissolution of both infection and cystine stones [26]. The course of calcium stone residuals is unpredictable, whereas some calcium stone fragments might provide a basis for new stone formation others will not [113]. Symptomatic patients should of course be subject to active stone removal, but in asymptomatic patients, long-term surveillance with appropriate recurrence prevention is recommended and is usually sufficient [15, 114, 115].

**Fig. 9:**
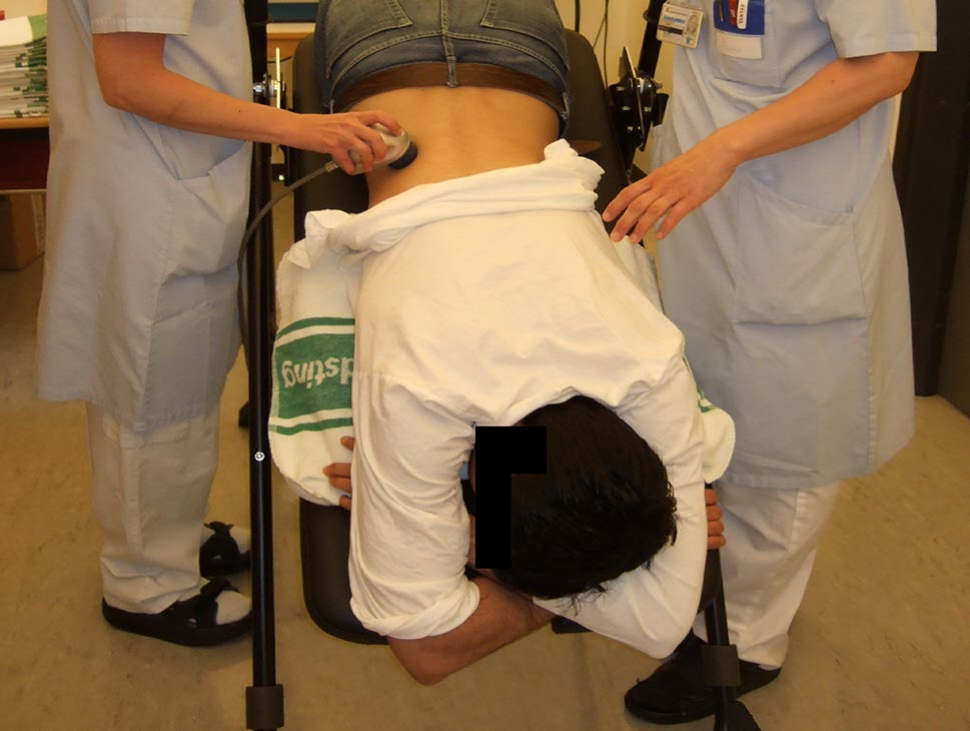
Inversion therapy (DIVE). High diuresis is combined with inversion and vibration over the kidney in attempts to move and eliminate fragments

To maintain a non-invasive therapeutic concept, percussion and inversion therapy is an option that should be considered. Several literature reports have shown that such a regimen can be used for improved elimination of residual fragments [116–118]. Not all authors have been successful with inversion therapy [119], and it seems necessary with standardization and improvement of this method to increase the rate of fragment clearance (Fig. 9). The advantage is that many patients treated with inversion therapy can be rendered stone free without invasive procedures. It is possible that elimination of residual fragments in the lower calyces in the future more effectively can be accomplished by ultrasound propulsion [120].

It is important not to forget, that also following meticulous clearing of the renal collecting system, a large proportion of patients still suffer the risk of recurrent stone formation [113, 121, 122]. When such a risk is pronounced, metabolic risk evaluation and recurrence preventive measures should be considered.

## Conclusion

From our more than 30-year experience with SWL, we have shown that this non- or low-invasive technique can be successfully used for treatment of a wide range of stone problems. The pre-requisites for success are to pay appropriate attention to the problems presented by the individual patient and accordingly adapt and optimize the therapeutic approach. With SWL, it is possible to offer the patient a non-invasive or least invasive method for stone removal without general or regional anaesthesia in an out-patient setting. Moreover, the complications can be kept at a low level and, not least important today, SWL is economically favourable compared with alternative methods. For every patient, the decision of the best treatment has to be a balance between the goal of the treatment and the efforts required to reach that goal.
